# Multi-omics study of mitochondrial dysfunction in the pathogenesis of hyperuricemia

**DOI:** 10.1080/0886022X.2025.2532855

**Published:** 2025-07-23

**Authors:** Yuechang Hong, Minghui Yang, Xin Xu, Peng Wang, Minqiang Fu, Renying Xiong, Jianjiang OuYang

**Affiliations:** a School of Clinical Medicine, Jiangxi University of Traditional Chinese Medicine, Nanchang, Jiangxi Province, People’s Republic of China; b Department of Sports Medicine, Affiliated Hospital of Jiangxi University of Traditional Chinese Medicine, Nanchang, People’s Republic of China; c Department of medicine, Affiliated Hospital of Jiangxi University of Traditional Chinese Medicine, Nanchang, People’s Republic of China

**Keywords:** Hyperuricemia, Mendelian randomization, mitochondrion, methylation, gene expression, protein

## Abstract

**Background:**

Mitochondrial dysfunction is linked to hyperuricemia (HUA), but its genetic pathophysiology is not yet fully understood. We employed Mendelian randomization (MR) to integrate multi-omics data and explore the associations between mitochondrial-related genes and HUA.

**Methods:**

We conducted a summary data-based MR analysis to investigate potential targets associated with HUA by integrating mitochondrial-related DNA methylation, gene expression, and protein quantitative trait loci. Additionally, to further explore the potential associations between DNA methylation, gene expression, and protein abundance, we performed MR and co-localization analyses to examine causal relationships between candidate gene methylation and expression, as well as between gene expression and protein abundance.

**Result:**

Through the integration of multi-omics evidence, we identified one primary gene, NUDT2, and three secondary genes, BOLA1, COMT, and HAGH. At the protein level, NUDT2 and COMT are negatively correlated with HUA risk, whereas BOLA1 and HAGH are positively correlated with HUA risk. Our analysis revealed a positive correlation between the methylation of cg06605933 in BOLA1 and its protein levels, which aligns with the negative effect of cg06605933 methylation on HUA risk. Additionally, we observed a positive correlation between NUDT2 gene expression and protein levels, confirming its beneficial effect on HUA risk. Strong co-localization support was found between the methylation of cg06605933 (PPH4 = 85.1%) in BOLA1 and protein abundance, as well as between NUDT2 gene expression (PPH4 = 96.6%) and protein levels

**Conclusion:**

Our study identified mitochondrial genes NUDT2, BOLA1, COMT, and HAGH as potentially associated with HUA risk, supported by evidence from various omics levels.

## Introduction

Hyperuricemia (HUA) is a metabolic disorder caused by purine metabolism abnormalities, leading to excessive production or reduced excretion of uric acid (UA). Gout is a consequence of the progression of HUA; when serum urate levels remain persistently elevated, urate becomes supersaturated and precipitates as needle-shaped crystals that deposit in the joints—especially in cooler or more vulnerable areas such as the great toe, ankle, and knee—and soft tissues, thereby triggering inflammation. This inflammatory response can result in gouty arthritis and related diseases [1]. While HUA is an essential prerequisite for gout, only a minority of individuals with HUA eventually develop gout [2]. Moreover, research has demonstrated that Cardiovascular disease, including hypertension, myocardial infarction and heart failure, are linked to elevated UA levels [3,4]. An epidemiological survey indicates that, as of 2020, the global prevalence of gout ranges from 1% to 6.8%, with the number of cases projected to exceed 120 million by 2035. This trend has positioned gout as one of the most common forms of inflammatory arthritis in Western countries. [5]. The global prevalence of gout has increased significantly, contributing to a substantial disease burden worldwide [6].

Research has demonstrated that xanthine oxidoreductase in the liver can enhance the production of reactive oxygen species (ROS) by generating superoxide and hydrogen peroxide. This process can lead to excessive ROS accumulation, which in turn may cause mitochondrial dysfunction [7–9].Additionally, studies suggest that uric acid may induce mitochondrial dysfunction and apoptosis through the downregulation of mitochondrial phosphatidylserine decarboxylase [10]. Genome-wide association studies (GWAS) have also identified several susceptibility variants. In European populations, GWAS estimates that the heritability of serum uric acid levels ranges from 45% to 68% [11]. Furthermore, research has shown that rats with hyperuricemia exhibit increased damage to mitochondrial DNA that is associated with elevated levels of intrarenal uric acid and oxidative stress [12]. Although the mechanisms underlying uric acid production and metabolism are relatively well understood, research into specific mitochondrial-related genes and their downstream effects on hyperuricemia remains limited.

Mendelian Randomization (MR) is a statistical method employed in epidemiological research to infer causality. Compared to observational studies, this approach is less susceptible to confounding and reverse causation biases, which enhances the reliability of causal inferences. This is because genes are randomly distributed during gamete formation, and genetic variation is determined at conception, remaining relatively stable throughout life, akin to random assignments in randomized experiments. This random distribution makes the relationship between genes and confounding factors relatively independent. It utilizes genetic variants that are strongly associated with exposure factors as instrumental variables to assess the causal relationships between these factors and outcomes. By mitigating the influence of confounding variables, MR provides a clearer understanding of the true effects of exposure on outcomes. This study combines data on mitochondrial DNA methylation, gene expression, and protein abundance to investigate the potential associations between mitochondrial gene methylation, expression, protein levels, and the risk of HUA using MR.

## Methods

### Research process

Figure S1 outlines the design process of this study. Initially, mitochondrial-related instrumental variables were selected from quantitative trait loci associated with DNA methylation, gene expression, and protein abundance. The dataset from Sakaue S served as the primary validation cohort. Subsequent analyses included SMR (Summary-data-based Mendelian Randomization) and HEIDI (Heterogeneity in Dependent Instrument) tests to further elucidate the causal relationships between exposures and outcomes. The FinnGen dataset provided additional validation, while tissue-specific data from the GTEx website offered further confirmation. Finally, by integrating MR analysis results across the three levels—methylation, gene expression, and protein abundance—the study aimed to identify candidate genes related to mitochondria involved in the pathogenesis of HUA.

### Data sources

Methylation quantitative trait loci (mQTL) data were derived from SNP-CpG associations in blood samples of 1,980 individuals of European ancestry, as reported by McRae et al. [13]. Blood expression quantitative trait loci (eQTL) data were obtained from the eQTLGen consortium, which includes samples from 31,684 individuals of European descent, covering 16,987 genes and 31,684 cis-eQTLs [14]. The protein quantitative trait loci (pQTL) dataset originated from the UK Biobank Pharmacogenomics Project (UKB-PPP), a comprehensive pQTL study involving 54,219 European individuals. This dataset provided summary statistics on genetic associations with 2,923 circulating proteins [15].

MitoCarta is a comprehensive catalog of genes encoding the mammalian mitochondrial proteome, assembled using a Bayesian integration of various sequence features and experimental datasets. Compared to version 2.0, version 3.0 excludes 100 genes and incorporates 78 new ones, resulting in an updated list of 1,136 human genes. After cross-referencing mitochondrial genes with different levels of QTL data, 2,560 genes associated with methylation, 864 genes related to gene expression, and 92 genes connected to protein abundance were identified.

The primary validation cohort for the genome-wide association study on HUA is derived from a study by Sakaue S et al. which encompasses 343,836 European individuals (https://www.ebi.ac.uk/gwas/, GWAS Catalog ID: GCST90018977). The GWAS data for the replication cohort were obtained from the Finngen database, which encompasses a study involving 384,093 individuals of European descent. Tissue-specific validation data were obtained from the GTEx portal (https://www.gtexportal.org/home/). Research suggests that the generation and metabolism of uric acid are primarily associated with the kidneys, liver, and small intestine [16,17]. Consequently, we utilized eQTL data from these relevant tissues to perform validation alongside the primary validation cohort (Table S1).

### Summary-data-based MR analysis

Summary-data-based MR (SMR) utilizes summary data from genome-wide association studies and eQTL studies to evaluate the pleiotropic associations between gene expression levels and complex traits. It employs genetic variants (single-nucleotide polymorphisms, SNPs) as instrumental variables to assess causal relationships between exposures and outcomes [18]. Compared to traditional MR approaches, SMR targets genes within a chromosomal window of ±1,000 kb and filters the most relevant cis-QTLs using a threshold *P*-value of 5.0 × 10^−8^, thereby enhancing statistical power. The HEIDI (heterogeneity in dependent instruments) test is used to determine whether phenotypic effects mediated by gene SNPs are attributable to linkage disequilibrium. SMR and HEIDI analyses were performed using the SMR software tool (SMR v1.3.1). The Benjamini-Hochberg method was employed to adjust P-values, controlling the false discovery rate (FDR) at α = 0.05. Association analyses were conducted with FDR-adjusted P-values < 0.05 and P*_HEIDI_* > 0.05.

### Colocalization analysis

Based on SMR analysis and HEIDI testing, we identified genes associated with mitochondrial methylation, gene expression, protein abundance, and HUA. To further investigate these associations at various molecular levels, we conducted colocalization analysis using the coloc package. Prior probabilities of 1 × 10^−4^ were assigned to hypotheses H1 and H2, and a prior probability of 1 × 10^−5^ was assigned to hypothesis H4, as per the default settings of the coloc.abf function. Colocalization evidence was deemed significant when applying the combined threshold (PPH4 ≥ 0.7), suggesting that the gene mediates a causal effect on HUA risk [18,19].

### Integrating results at the multi-omics level of evidence

To investigate the impact of mitochondrial-related molecular layers on HUA, we integrated analyses across three levels: methylation, gene expression, and protein abundance. Given that proteins are the ultimate products of gene expression, we focused on identifying candidate genes that show associations with HUA specifically at the protein abundance level. Based on this criterion, we classified the final candidate genes into two tiers. Tier 1: Genes in this tier were associated with HUA at the protein abundance level (FDR-corrected P-value < 0.05, P *_HEIDI_* > 0.05) and also showed associations with HUA at both the gene methylation and expression levels (FDR-corrected P-value < 0.05, P *_HEIDI_* > 0.05). Tier 2: Genes in this tier were associated with HUA at the protein abundance level (FDR-corrected P-value < 0.05, P *_HEIDI_* > 0.05) and also showed associations with HUA at either the gene methylation or expression level (FDR-corrected P-value < 0.05, P *_HEIDI_* > 0.05). Furthermore, to explore the mechanisms underlying these associations between mitochondrial-related molecular layers, we conducted Mendelian randomization (MR) and colocalization analyses. These analyses aimed to investigate the causal relationships between mitochondrial DNA methylation and gene expression, as well as between gene expression and protein abundance.

### Ethics

The GWAS, mQTL, eQTL, and pQTL data utilized in this study are publicly available summary-level datasets; therefore, no additional ethical approval or informed consent is required.

## Results

### Mitochondrial gene methylation and HUA

In this study, we identified a total of 487 mitochondrial-related CpG sites associated with the risk of hyperuricemia. Following FDR correction (*p* < 0.05) and the HEIDI test (P-HEIDI > 0.05), we ultimately pinpointed 95 CpG sites linked to 57 distinct genes (Table S2). For example, the CpG site cg20709110 in COMT (OR 0.986, 95% CI 0.977–0.994) was negatively associated with HUA risk, whereas cg06605933 in BOLA1 (OR 1.022, 95% CI 1.008–1.036) showed a positive association. Furthermore, cg00935504 in HAGH (OR 0.993, 95% CI 0.990–0.997) and cg08240373 in NUDT2 (OR 1.008, 95% CI 1.003–1.014) were also positively associated with HUA risk. The CpG sites cg17724175 in MCL1, cg08815677 in NDUFA13, and cg04439028 in TRAP1 were successfully replicated in FinnGen (Table S3).

### Mitochondrial gene expression and HUA

A total of 192 mitochondrial-related gene expression sites were identified as being associated with the risk of HUA. After applying FDR correction (*p* < 0.05) and the HEIDI test (P *_HEIDI_* > 0.05), we identified 29 unique genes significantly associated with HUA risk (Table S4). Among these genes, the following exhibited a significantly negative association with HUA risk: FXN (OR 0.862, 95% CI 0.788–0.943), MDH2 (OR 0.908, 95% CI 0.857–0.963), SLC25A44 (OR 0.902, 95% CI 0.857–0.949), MRPS30 (OR 0.925, 95% CI 0.877–0.975), NDUFB6 (OR 0.934, 95% CI 0.893–0.977). Conversely, there were 14 genes demonstrated a significantly positive association with HUA risk (Figure S2). Notably, MFF was confirmed in the FinnGen study (Table S5).

### Mitochondrial protein and HUA

A total of 15 mitochondrial-related proteins were identified as being associated with HUA risk. Following FDR correction (*p* < 0.05) and the HEIDI test (P *_HEIDI_* > 0.05), 7 of these proteins were found to be significantly linked to HUA risk (Table S6). Among them, the following proteins were negatively associated with HUA risk: COMT (OR 0.99, 95% CI 0.979–0.995), NUDT2 (OR 0.98, 95% CI 0.966–0.991), and SOD2 (OR 0.96, 95% CI 0.942–0.988). Conversely, BOLA1 (OR 1.06, 95% CI 1.023–1.106), DCXR (OR 1.10, 95% CI 1.052–1.16), HAGH (OR 1.07, 95% CI 1.029–1.117), and SDHB (OR 1.19, 95% CI 1.099–1.296) were positively associated with HUA risk (Figure S3). Although MR analysis in the FinnGen study did not identify a causal relationship between any genetic predictors of protein abundance and HUA risk, most associations retained a consistent direction (Table S7).

### Integrating multi-omics evidence

Through the integration of multi-omics evidence, we identified one primary gene, NUDT2, and three secondary genes, BOLA1, COMT, and HAGH (Table S9). At the protein level, NUDT2 and COMT are negatively correlated with HUA risk, whereas BOLA1 and HAGH are positively correlated with HUA risk. Our analysis revealed a positive correlation between the methylation of cg06605933 in BOLA1 and its protein levels, which aligns with the negative effect of cg06605933 methylation on HUA risk. Additionally, we observed a positive correlation between NUDT2 gene expression and protein levels, confirming its beneficial effect on HUA risk. Strong co-localization support was found between the methylation of cg06605933 in BOLA1 and protein abundance, as well as between NUDT2 gene expression and protein levels (Table S8). Figure S4 displays a Manhattan plot illustrating the associations between mitochondrial-related gene molecular features and HUA.

### Tissue-specific validation

Serum uric acid levels are closely associated with the liver, kidneys, and small intestine. Consequently, we further investigated the impact of the identified genes on HUA in specific tissues. Our analysis revealed that, among the candidate genes, only NUDT2 expression levels exhibited tissue specificity. Gene-predicted NUDT2 expression was linked to a reduced risk of HUA in the sigmoid colon (OR 0.991, 95% CI 0.986–0.995, P*_FDR_* 0.003), transverse colon (OR 0.991, 95% CI 0.987–0.995, P *_FDR_* 0.002), ileum (OR 0.989, 95% CI 0.982–0.994, P *_FDR_* 0.004), and liver (OR 0.991, 95% CI 0.985–0.995, P *_FDR_* 0.007).

## Discussion

To the best of our knowledge, this study is the first to employ multi-omics approaches to explore the potential mechanisms linking mitochondrial dysfunction with HUA. Utilizing SMR and HEIDI tests, we investigated the associations between mitochondrial gene methylation, expression, and protein abundance with HUA. Our findings revealed associations between mitochondrial genes NUDT2, COMT, BOLA1, and HAGH and HUA, supported by evidence from multiple omics layers. Further integration of these omics data and co-localization analysis reinforced these results. Additionally, using the same methodology, we identified an association between NUDT2 and HUA in specific tissues, which further strengthens the reliability of the study.

Among the three aspects of mitochondrial gene methylation, expression, and protein abundance, NUDT2 is found to be associated with all of them. Nudix hydrolase 2 (NUDT2) hydrolyzes diadenosine 5′,5′′′-p1,p4-tetraphosphate (Ap4A) to generate AMP and ATP [20]. The production of AMP subsequently enhances the activity of AMP deaminase, converting AMP into inosine monophosphate [21]. Recent studies have demonstrated that NUDT2 is closely linked to breast cancer, intellectual disabilities, and peripheral neuropathy [22–24]. Additionally, uric acid-induced endothelial dysfunction is associated with decreased mitochondrial quality and reduced ATP production [12]. Xanthine oxidoreductase can lead to the release of hypoxanthine as xanthine or uric acid from cells before IMP is restored, thereby affecting blood uric acid levels [25]. The association between oxidative stress regulators and hyperuricemia highlights the importance of considering xanthine oxidase overactivation in addition to hyperuricemia alone [4]. Moreover, previous epidemiological studies have established a strong association between hyperuricemia and neuroprotection [26]. Therefore, we hypothesize that Nudix hydrolase 2, encoded by NUDT2, is significantly associated with HUA, although further experimental validation is required. Current multi-omics research supports this hypothesis and further suggests that NUDT2 expression is negatively correlated with HUA risk through increased NUDT2 protein abundance, with additional co-localization analysis reinforcing this finding.

BOLA1 is an aerobic mitochondrial protein that plays a crucial role in oxidative stress by preventing mitochondrial morphological changes induced by glutathione [27]. Research has demonstrated that oxidative stress levels in individuals with type 2 diabetes and obesity can significantly affect uric acid concentrations. Uric acid is recognized as a key antioxidant that can mitigate oxidative damage in obese individuals [28]. Our study reveals that genetically predicted BOLA1 methylation is associated with HUA through its impact on protein abundance. Specifically, the methylation of cg06605933 is positively correlated with BOLA1 protein levels and is strongly supported by colocalization evidence. Additionally, COMT encodes catechol-O-methyltransferase, which is predominantly expressed in the liver and kidneys. It plays a critical role in regulating dopamine activity in the kidneys [29,30]. Previous studies have established a link between uric acid and urate excretion and dopamine-induced glomerular filtration [31]. Our MR analysis provides evidence that methylation of cg20709110 is negatively correlated with both protein abundance and HUA. Additionally, we identified rs4680 as the top SNP associated with HUA within COMT. This finding aligns with previous research showing that the missense variant rs4680 (G > A) reduces gout risk [32]. ur results suggest that methylation of cg20709110 may enhance COMT protein abundance, potentially lowering HUA risk; however, further research is necessary to confirm these findings.

HAGH, also known as methylglyoxalase-2, is involved in regulating oxidative stress. Elevated levels of methylglyoxal in plasma, under certain conditions, can lead to the production of ROS and contribute to oxidative stress [33]. It has been reported that plasma levels of HAGH protein are associated with Alzheimer’s disease in carriers of apolipoprotein E [34]. However, its association with HUA remains underexplored. Our study reveals that both HAGH methylation and protein abundance are associated with HUA risk. Specifically, methylation of cg00935504 is negatively correlated with HAGH protein levels, a finding supported by colocalization evidence.

Through MR analysis, we investigated the causal relationship between mitochondrial gene methylation, expression, and protein abundance associated with HUA. Additionally, we integrated findings from multi-omics evidence, further strengthening the association between mitochondrial-related genes and HUA risk. Furthermore, the large sample size from the GWAS significantly enhanced the statistical power of our study. However, we encountered several limitations. Our reliance on QTL data to examine the relationship with HUA may have overlooked other potential pathogenic factors. Moreover, this study primarily involved individuals of European ancestry, which may introduce biases due to variations in region, ethnicity, gender, or age. This variability indicates that the influence of diverse ethnicities and regions on our findings cannot be disregarded, and caution should be exercised when interpreting the causal relationship between mitochondrial-related genes and HUA risk in other populations. Lastly, all data and results in this study were derived from non-experimental assessments, lacking experimental verification, which necessitates further validation through randomized controlled trials.

## Conclusion

In summary, our research integrates mitochondrial-related DNA methylation, gene expression, and protein abundance to reveal potential causal relationships with HUA, highlighting the importance of mitochondrial-related genes NUDT2, BOLA1, COMT, and HAGH in the pathogenesis of HUA. These findings deepen our understanding of the pathology of HUA and aid in identifying potential drug targets.

## Supplementary Material

supplementary document.xlsx
